# Supplementary material for: Chimeric Antigen Receptor T Cell Bearing Herpes Virus Entry Mediator Co-Stimulatory Signal Domain Exhibits Exhaustion-Resistant Properties
Source: Int J Mol Sci. 2024 Aug 8;25(16):8662. doi: 10.3390/ijms25168662 (PMC11354286; doi:10.3390/ijms25168662)
Supplement: Supplementary file 1 [file ijms-25-08662-s001.zip › 08-08-24 Suppl Figure.pdf]

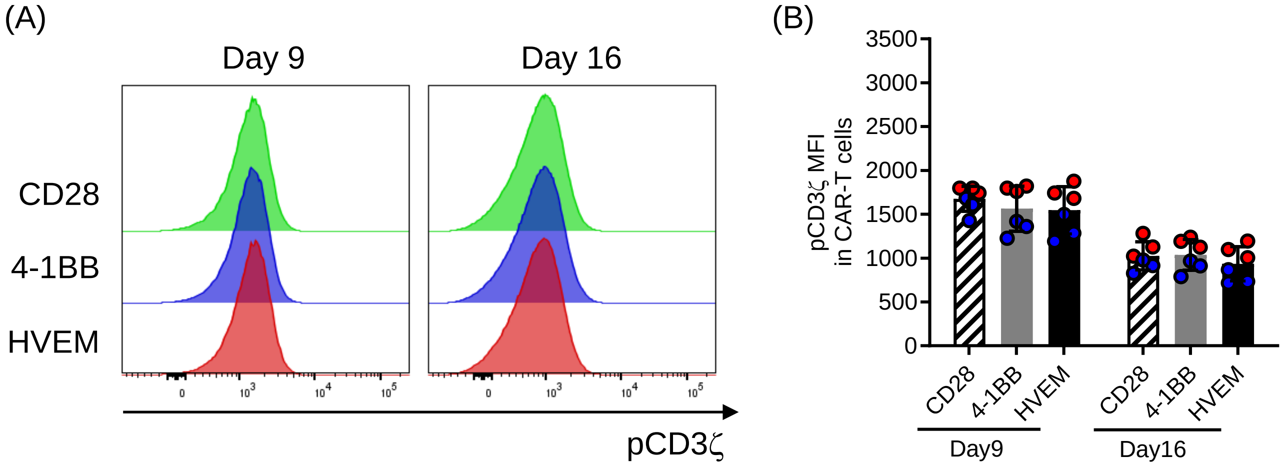

Figure S1. Analysis of CD3ζ phosphorylation in non-transduced (GFP⁻) population of vector-transduced T cells. (A) Panels show typical phosphorylated CD3ζ histogram of GFP⁻ populations in the CAR-T cells with different CSSD (CD28, 4-1BB or HVEM) at day 9 (left) and 16 (right) analyzed by flow cytometry. (B) Bar graphs show the MFI of phosphorylated CD3ζ in the GFP⁻ populations of the CAR-T cells with different CSSD at day 9 (left) and 16 (right). Each bar shows the mean with SDs with each data point as filled circle. The results from two separate experiments with primary $CD8^+$ T cells from two different donors (*n* = 6) are shown in red or blue filled circles.
